# Classifying Ectopia Lentis in Marfan Syndrome into Five Grades of Increasing Severity

**DOI:** 10.3390/jcm9030721

**Published:** 2020-03-06

**Authors:** Jean-Christophe Zech, Audrey Putoux, Evelyne Decullier, Anne-Emmanuelle Fargeton, Patrick Edery, Henri Plauchu, Sophie Dupuis-Girod

**Affiliations:** 1 Centre Ophtalmologique Kleber, 69006 Lyon, France; christophezech@gmail.com; 2 Service de Génétique, Unité de Génétique Clinique, Centre Labellisé Anomalies du Développement, Hospices Civils de Lyon, 69500 Bron, France; audrey.putoux@chu-lyon.fr (A.P.); patrick.edery@chu-lyon.fr (P.E.); 3 Centre de Recherche en Neurosciences de Lyon, Equipe GENDEV, INSERM U1028, UMR CNRS 5292, Université Claude Bernard Lyon 1, 69500 Bron, France; 4 Unité de Recherche Clinique, Pôle Santé Publique, Hospices Civils de Lyon, 69003 Lyon, France; evelyne.decullier@chu-lyon.fr; 5 Service de Génétique, Unité de Génétique Clinique, Centre de Compétence Syndrome de Marfan et Apparentés, Hospices Civils de Lyon, 69500 Bron, France; henryplauchu@gmail.com (H.P.); sophie.dupuis-girod@chu-lyon.fr (S.D.-G.)

**Keywords:** ectopia lentis, Marfan syndrome, classification

## Abstract

Purpose: To describe a five-grade classification of ectopia lentis in Marfan syndrome (MFS) and to evaluate the positive predictive value of the early grades of ectopia lentis. Methods: We prospectively included MFS patients and their healthy relatives. The anterior segment examination was classified into grades 0 to 5, and we studied the sensitivity, specificity, and positive predictive value of ectopia lentis in this classification. Results: Seventy-four MFS patients and thirty-six healthy controls were examined. In the MFS group, grades 1, 2, 3, and 4 were present in 15, 24, 17, and 7 patients, respectively, whereas 11 patients in this group did not present ectopia lentis. In the control group, grades 0 and 1 were observed in 30 and 6 individuals, respectively. Sensitivity to ectopia lentis of at least grade 2 was 64.9%, with 100% specificity, whereas sensitivity to ectopia lentis of at least grade 1 was 85.1%, with 83.3% specificity. The positive predictive value of ectopia lentis that was greater than or equal to grade 2 was 100%, whereas that of ectopia lentis greater than or equal to grade 1 was 91.3%. Conclusions: High positive predictive values s were found to be associated with grades 2 and higher of the five-grade classification of ectopia lentis. This classification should help to harmonize clinical practices for this major feature of MFS.

## 1. Introduction

Marfan syndrome (MFS, OMIM #154700) is an autosomal dominant disorder with an estimated prevalence of one in 5000 individuals [1]. It is characterized by musculoskeletal manifestations, cardiovascular disease, and ocular abnormalities, particularly ectopia lentis [2,3]. MFS is usually caused by de novo or inherited mutations in the fibrillin-1 gene (*FBN1, MIM No 134797*) [4]. Diagnosis of MFS depends on clinical evaluation, family history, and molecular data, in accordance with the Ghent criteria [5], which were revised in 2010 [6]. However, direct sequencing of the *FBN1* gene detects mutations in only 70%–90% of cases [7,8].

*FBN1* is located on chromosome 15q12 and encodes the protein fibrillin, an essential component of the extracellular matrix [9]. *FBN1* mutations cause abnormalities in the incorporation of fibrillin into the connective tissue matrix [10]. Microfibrils are known to have an anchoring function in certain tissues, as is the case in the ciliary processes in the eye. The pathogenesis of the ocular abnormalities in MFS is poorly understood [11]. Fibrillin expression is important in the lens capsule and zonules, the ciliary processes, and the ciliary body [12]. In MFS, abnormal fibrillin fibers [13] and proteolytic degradation of fibrillin-rich microfibrils [14] have been suggested as possible etiologies for ectopia lentis [12]. Even if we are not able to predict the phenotype for any given *FBN1* mutations, ectopia lentis has been reported more frequently in cysteine missense mutation [15,16,17,18].

Ectopia lentis is the most common ocular manifestation in MFS with *FBN1* mutation (as described in the seminal paper by Maumenee in 1981 [3]) and is relatively specific to this disease when associated with other features. It is characterized by partial or complete displacement of the lens as a result of disruption to the zonular fibers. Patients report decreased or fluctuating vision. MFS ocular complications include cataract, myopia, glaucoma, amblyopia, corneal flatness, and retinal detachment [2,19]. Approximately 67% or 68% of cases of ectopia lentis are related to MFS [20]. Other etiologies of ectopia lentis include other hereditary systemic diseases such as homocystinuria (CBS, MIM 236200).

In MFS, ectopia lentis is typically bilateral and dislocated superior-temporally [11]. It is also considered to be one of the cardinal clinical features in the revised Ghent criteria [6]. However, clinical examinations for identifying ectopia lentis have not really been codified.

We report the ophthalmological examinations of 74 patients, with a diagnosis of MFS confirmed by Sanger sequencing *FBN1* analysis, and 36 controls. The purpose of this study was to describe a 5-grade classification of increasing severity for ectopia lentis based on clinical examination and to evaluate the predictive value for the early grades of ectopia lentis in order to help characterize this major clinical diagnosis criterion.

## 2. Experimental Section

We prospectively performed ocular examinations of MFS patients seen between 2000 and 2013 in the Marfan syndrome expert centre located in Lyon, as well as of their relatives without MFS (controls).

The patients had a clinical diagnosis of MFS according to the revised Ghent criteria (ectopia lentis was not taken into account for the diagnosis), confirmed by *FBN1* sequencing. The controls were relatives of the MFS patients with none of the clinical features of MFS and in whom testing for the familial *FBN1* mutation by Sanger sequencing was negative.

All examinations were performed by the same operator (J.-C.Z.), an expert ophthalmologist working at the Marfan syndrome expert center. Pupillary dilation was performed according to a strict and reproducible protocol. Tropicamide and phenylephrine were instilled every five minutes, three times. Ocular examinations were carried out twenty minutes after the last instillation. After maximal pupillary dilation, the ophthalmological examination included visual acuity measurement with and without correction; ophthalmoscopy using a slit lamp biomicroscopy to analyze the anterior segment; and a three-mirror lens to appreciate the quality of the dilation validated by the absence of pupillary reflex and to search for ectopia lentis. The examination was performed in primary gaze, downward gaze, and lateral gaze (15–25°).

**Figure 1:**
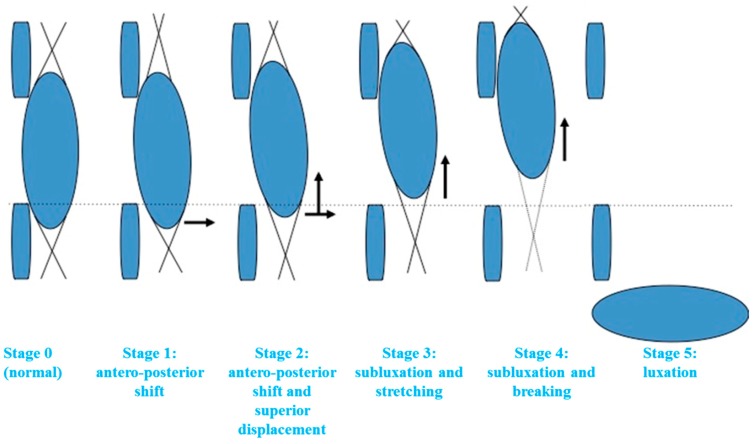
Progressive grades of lens dislocation in Marfan syndrome (MFS) (complete description in Table 1).

**Figure 2:**
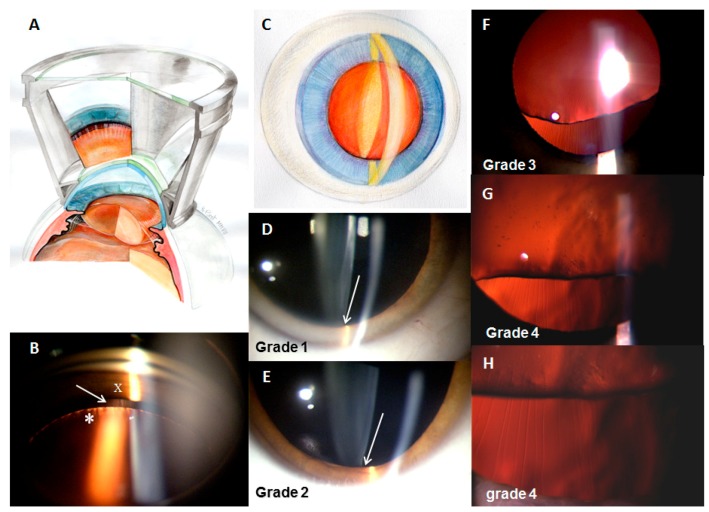
Slit lamp and three-mirror lens examination in MFS. (**A**) Schematic representation of the three-mirror lens in MFS. The three-mirror lens is placed between the slit lamp and the patient’s eye to search for lens dislocation. (**B**) Visualization through a mirror with the three-mirror lens of a space (arrow) between the iris (X) and the lens (*), which can correspond to a grade 2. (**C**) Schematic representation of the result of a slit lamp examination, showing a shift in the vertical light beam between its impact on the iris and the anterior side of lens. (**D**) Grade 1: we observe a minimal shift in the vertical light beam between its impact on the iris and the anterior side of lens. (**E**) Grade 2: we observe a greater shift in the vertical light beam between the iris and the lens. (**F**–**H**) From grade 3, using retro-illumination of retinal reflect by positioning the slit lamp in a central position makes precise visualization of the inferior part of the lens possible, as well as detailed analysis of the density of the zonular fibers. In grade 3 (**F**), the inferior part of the lens is clearly visible in primary gaze. Zonular fibers are considerably stretched but not broken. The density of the fibers appears normal. In grade 4 (**G**,**H**), the zonular fibers are broken, and the density of the fibers is reduced. The inferior part of the lens is crenellated. The few unbroken fibers appear to be less strained and adopt a wavy appearance. The density of the zonular fibers decreases. The inferior side of the lens becomes irregular because of modification to the zonular tension.

Five grades were defined to describe an abnormal lens examination (see Figure 1 and Figure 2; Table 1).

Only the results of the examinations with optimal dilation were entered into the database for this study. In cases involving different grades in the right and left eyes, the highest grade was taken into consideration. Patients who had surgery for ectopia lentis were not included in the cohort.

To evaluate whether or not early grades of ectopia lentis were correlated with the presence of MFS, we compared the diagnostic values (sensitivity, specificity, positive predictive value (PPV), and negative predictive value (NPV)) of ectopia lentis by testing three thresholds: from grade 1, from grade 2, and from grade 3. Exact confidence intervals were presented for each parameter.

## 3. Results

The characteristics of the patients and controls are summarized in Table 2. A total of 110 individuals (74 MFS patients and 36 controls) were enrolled in this study. They were classified according to the ophthalmological examination results on the basis of pre-defined grades. The results are summarized in Table 3.

No incidence of grade 5 was observed in our cohort. All the patients are being closely monitored, in particular with regard to ophthalmological features, and surgery is performed before grade 5 is attained.

The results for sensitivity, specificity, and predictive values are summarized in Table 4. In addition, evolution in ocular manifestations has been collected for the MFS patients included in our cohort, and are detailed in Table 5.

## 4. Discussion

Ectopia lentis is a major clinical diagnosis criterion for MFS, but when the displacement of the lens is small, the threshold for retaining this diagnosis criterion has not been defined. However, it is important to correctly identify the presence of this criterion because the diagnosis of MFS can be retained even in the absence of the *FBN1* mutation, if the clinical diagnostic criteria are satisfied. On the other hand, the diagnostic criteria must be stringent in order to prevent misdiagnosis of Marfan syndrome.

Ectopia lentis grading systems have been described previously by Chandra [21] in order to classify subluxation according to the direction and extent of lens movement, and by Waiswol [22] in order to assess a predictive value for surgical outcomes. Neither of these interesting categorizations are specific to MFS, and they are not helpful for evaluating this clinical criterion for diagnostic purposes. In 2003, M. De Saint Jean [23] presented a study of 198 patients and concluded that the presence of ectopia lentis of greater than or equal to 1 mm had a high predictive value for diagnosis of MFS. They classified ectopia lentis into four categories: absent, mild (<1 mm, detected as visibility of inferior zonules or of the inferior edge of the lens), moderate (= 1 mm), or severe (>1 mm). This subdivision did not describe the early grades precisely: the mild grade effectively includes both visualization of the equatorial part of the lens (grade 3 in our classification) and visualization of inferior zonular fibers (grade 2 in our classification).

This study highlighted the usefulness of the three-mirror lens in the diagnosis of early grades of EL. To ensure reproducibility, ocular examinations must include all the steps described in the methods. To identify lens displacement, a lateral gaze (15–25°) slit lamp biomicroscopy is essential for accurate analysis of the relief of the anterior segment of the eye and visualization of a very small space between the iris and the lens. In grades 1 and 2, this space is so small that you can only see it if you search for it specifically. In grades 3 and higher, the use of retro-illumination through the retinal reflect by positioning the slit lamp in the central position made visualization of the equatorial part of the lens possible, as well as the density of the zonular fibers. The three-mirror lens can be useful for a more precise observation; it makes a fundus examination possible, and can thus confirm the presence of a space between the iris and the lens in grades 1 and 2. There is sometimes a protrusion of the anterior part of the iris, leading to visualization of a space between the iris and the lens during the slit lamp biomicroscopy. The three-mirror lens shows that the iris is in contact with the lens, confirming the absence of any anomaly suggestive of MFS. Another method to visualize the inferior edge of the lens and the inferior zonules when looking for posterior and superior displacement of the lens could be to use retroillumination of the lens while the patient is looking down [24].

In children, the pupil dilation influences biometric parameters, including lens position [25]. When dilation is excessive, it can lead to observation of a minimal space in the inferior position, between the posterior surface of the iris and the anterior wall of the equatorial part of the lens, and this result could be interpreted as grade 1. In the same way, visibility of inferior zonules is closely related to pupillary dilation. The degree of pupillary dilation must be taken into consideration for interpreting grades 1 and 2.

As expected, the results of this study show that grades 3 or higher are specific to EL, but the sensitivity of these grades is relatively low (32%). Taking grades 2 or higher into consideration increased the sensitivity (65%) and negative predictive value for ectopia lentis (58%) without decreasing the specificity (100%) and positive predictive value (100%). Considering grades 1 or higher made possible a significant increase in the sensitivity (78%) and negative predictive value (69%), but led to a decrease in the specificity (83%) and positive predictive value (91%). We therefore suggest considering ectopia lentis as a criterion for Marfan from grade 2 onward.

In the MFS group in our cohort, 65% of the patients had ocular manifestations (ectopia lentis grade 2 and higher), which is consistent with the data in the literature. The follow-up of these patients is closely monitored if grade 3 or higher has been observed. However, patients (especially adults) with ectopia lentis at an early grade (up to grade 2) do not have regular controls. In our cohort, ectopia lentis grades evolution was observed in 22% of MFS patients.

A limitation of this study was the single-center examinations. This method was chosen to ensure reproducibility for establishing the description, but reproducibility with several operators has not been performed. Furthermore, the data on follow-up of ophthalmic criteria are not complete for early grades; it would have been interesting to study the proportion of patients with unfavorable progression in this cohort.

## 5. Conclusions

To conclude, we suggest using this 5-grade classification for describing ectopia lentis in MFS. It has the advantage of providing a precise description of the degrees of lens displacement with a defined method that can be used by all ophthalmologists. It should help harmonize clinical practices for this major feature of MFS and will be useful for clinical diagnosis of patients with ophthalmological features at early grades. The ophthalmological examination must include measurement of visual acuity with and without correction, ophthalmoscopy using a slit lamp biomicroscopy, and a three-mirror lens. The exam must be done in primary and downward gaze, as described.

We showed that grades 2 and higher have a high PPV for ectopia lentis and a clinical diagnosis of MFS.

For grade 1, we suggest considering it as a potential feature and re-evaluating patients 1 year later. As this grade probably corresponds to the beginning of the dislocation, monitoring patients with grade 1 may help to significantly reduce delays in clinical diagnosis when the ascending aorta is dilated, and lead to faster diagnosis of late or mild forms.

## Figures and Tables

**Table 1:** Description of the different grades observed at ocular examination.

	Eye in Primary Position	Gaze Directed Down
**Grade 1** (Figure 2D) **Minimal shift**	Anteroposterior shift in the inferior equatorial part of the lens resulting in a minimal space in an inferior position, between the posterior surface of the iris and the anterior wall of the equatorial part of the lens. No visualization of the equatorial zonular fibers.	Visualization of a space and sometimes of some zonular fibers
**Grade 2 ** (Figure 2E) **Shift and superior displacement**	Anteroposterior displacement of the inferior part of the lens was associated with its superior displacement. Peripheral adherence of the zonule on the inferior part of the lens became visible, whereas it is normally masked by the iris. The inferior part of the lens was not visible in primary gaze.	Visualization of the equatorial part of the lens
**Grade 3** (Figure 2F) **Subluxation and stretching**	Evident superior displacement of the lens with visualization of the equatorial part. Inferior zonular fibers are stretched but not broken, well seen on direct retina reflection. The density of fibers appears normal. Visual acuity can be deteriorated.	–
**Grade 4** (Figure 2G,H) **Subluxation and breaking**	Breaking of some zonular fibers; the few unbroken fibers adopt a wavy appearance. Density of the fibers decrease. Inferior side of the lens becomes irregular because of modification of zonular tension. We were sometimes able to observe phacodonesis with the ocular movements.	–
**Grade 5** **Total luxation**	Lens can be visualized in the vitreous, at the peripheral inferior region.	–

**Table 2:** Patient’s characteristics.

	Overall	MFS Group	Control Group
**Number (n)**	110	74	36
**Sex (M/F)**	56/54	39/35	17/19
**Mean age at examination**	–	25.3 (3.8–54.8)	38.5 (7.9–64.4)

M, male; F, female.

**Table 3:** Number of MFS patients and controls classified into each grade described in Table 1.

Grade	MFS Group n = 74 (%)	Mean Age at Examination (Min–Max)	Control Group n = 36 (%)	Mean Age at Examination (Min–Max)
Normal (0)	11 (14.8)	35 (14–55)	30 (83.3)	30 (8–64)
1	15 (20.3)	28 (7–52)	6 (16.7)	21 (15–40)
2	24 (32.4)	25 (6–46)	0 (0)	–
3	17 (23.0)	19 (6–47)	0 (0)	–
4	7 (9.5)	20 (4–31)	0 (0)	–
5	0 (0)	–	0 (0)	–
Total EL	63 (85.1)	23 (4–52)	6 (16.7)	21 (15–40)

EL, ectopia lentis.

**Table 4:** Results of sensitivity, specificity, positive predictive value, and negative predictive value of ocular examination.

	Sensitivity		Specificity		Predictive Values	
Thresholds	n	% (CI *)	n	% (CI *)	Positive % (CI *)	Negative % (CI *)
≥grade 3	24/74	32.4 (22–44.3)	36/36	100 (90.3–100)	100 (85.8–100)	41.9 (31.3–53)
≥grade 2	48/74	64.9 (52.9–75.6)	36/36	100 (90.3–100)	100 (92.6–100)	58.1 (44.8–70.5)
≥grade 1	63/74	85.1 (75–92.3)	30/36	83.3 (67.2–93.6)	91.3 (82–96.7)	73.2 (57.1–85.8)

* confidence interval.

**Table 5:** Evolution of ocular manifestations in MFS patients.

Initial Grade	Number of Patients (Percentage) with Follow Up in the Center	Ophthalmic Evolution Number of Patients (Age at Initial Examination) and Evolution
Grade 0 (n = 11)	n = 6 (54%)	3 patients (14, 41, 43 yo) = no change (3, 7, and 10 years after initial examination)
		1 patient (55 yo) = glaucoma
		1 patient (14 yo) = grade 1 (9 years after initial examination)
		1 patient (17 yo) = grade 2 (10 years after initial examination)
Grade 1 (n = 15)	n = 10 (67%)	7 patients (7 to 30 y) = no change (2 to 7 years after initial examination)
		1 patient (52 yo) = grade 2 (5 years after initial examination)
		1 patient (18 yo) = grade 3 (5 years after initial examination)
		1 patient (48 yo) = EL surgery (following a chock, 5 years after initial examination)
Grade 2 (n = 24)	n = 14 (58%)	12 patients (6 to 35 yo) = no change (1 to 12 years after initial examination)
		2 patients (20, 22 yo) = grade 3 (6 years after initial examination)
Grade 3 (n = 17)	n = 17 (100%)	12 patients (6 to 33 yo) = no change (2 to 8 years after initial examination)
		1 patient (47 yo) = cataract (3 years after initial examination)
		1 patient (9 yo) = grade 4 (8 years after initial examination)
		1 patient (17 yo) = RD surgery (2 years after initial examination)
		2 patients (13, 16 yo) = EL surgery (1 and 2 years after initial examination)
Grade 4 (n = 7)	n = 7 (100%)	3 patients (25, 28, 30 yo) = no change (4, 6, and 9 years after initial examination)
		2 patients (4, 17 yo) = EL surgery (1 year after initial examination for both)
		2 patients (7, 31 yo) = RD surgery (4 and 6 years after initial examination)

EL, ectopia lentis; RD, retinal detachment; yo, year old.
